# Hearing Loss in Young and Middle-Aged Adults as a Modifiable Risk Factor for Late-Life Dementia: A Systematic Review and Meta-Analysis

**DOI:** 10.3390/audiolres15060174

**Published:** 2025-12-12

**Authors:** Lakshmi Satheesan, Usha Shastri, Gagan Bajaj, Mohan Kumar Kalaiah

**Affiliations:** Department of Audiology and Speech Language Pathology, Kasturba Medical College Mangalore, Manipal Academy of Higher Education, Manipal 576104, Indiagagan.bajaj@manipal.edu (G.B.)

**Keywords:** young, middle-aged, hearing loss, cognitive decline, dementia risk

## Abstract

Background: Individuals with untreated hearing loss often experience cognitive decline as a result of increased cognitive load and reduced sensory stimulation. Despite the well-established link between untreated hearing loss and cognitive decline in older adults, its impact on cognition in young and middle-aged adults has not been systematically examined. Given the Lancet Commission’s identification of midlife hearing loss as the leading modifiable risk factor for dementia, early identification of cognitive decline is essential. This review explored the cognitive impact of untreated hearing loss in adults. Method: A comprehensive search was conducted in PubMed, Scopus, Web of Science, and EMBASE to include studies comparing cognitive function between adults with normal hearing and those with untreated hearing loss aged 18–65 years. The methodological quality of the included studies was examined via the Joanna Briggs Institute Critical Appraisal Checklist for Analytical Cross-Sectional Studies. Pooled mean differences and heterogeneity were analysed for each domain. Results: Seven studies included in the qualitative synthesis had “moderate” to “strong” methodological quality. The cognitive domains assessed in these studies were global cognitive function, memory, attention, and executive function. Of these, six were eligible for meta-analysis, which revealed a small but statistically significant decline in overall cognitive performance and memory and executive function among adults with untreated hearing loss. Conclusions: Cognitive vulnerabilities exist in young and middle-aged adults with untreated hearing loss. Hence, incorporating cognitive assessment into routine audiological evaluation may enable earlier intervention and delay the future burden of Alzheimer’s disease and related dementias in such a population.

## 1. Introduction

Nearly 2.5 billion people in the world suffer from hearing loss, 700 million of whom require hearing rehabilitation [1]. According to the World Health Organization [1], over 430 million people in the world’s population are left untreated for hearing loss. Untreated hearing loss and cognitive impairments often coexist and have a mutual impact on one another [2,3]. Individuals with hearing impairment may experience cognitive decline due to a lack of stimulus input and neural network activity, leading to reduced performance in cognitive–linguistic processes [4]. Many researchers have linked hearing loss with cognitive decline to understand its extent of impact on cognition [2,5]. Several hypotheses have been proposed to explain this link, including the sensory deprivation hypothesis, which suggests that reduced auditory input negatively impacts brain structure and function, leading to cognitive decline [6,7]. Similarly, the information degradation hypothesis posits that the increased cognitive effort required to process degraded auditory signals may divert cognitive resources from other functions [6]. Another perspective, the common-cause hypothesis, suggests that hearing loss and cognitive decline may share underlying factors such as neurodegeneration or vascular pathology [6,7]. A review by Loughrey et al. [8] revealed a statistically significant association between age-related hearing loss and cognitive domains, including executive functions, semantic memory, episodic memory, processing speed and visuospatial ability. A recent meta-analysis highlighted the increased risk of incident cognitive decline with adult-onset hearing loss [9].

Presbycusis is a gradually progressive disorder of aging, with degenerative changes beginning as early as young adulthood [10]. As a result, early detection of individuals at risk of cognitive decline is an important focus in healthcare settings [11]. This highlights that the association between hearing loss and an increased risk of cognitive decline is not confined to older adults but also affects younger and middle-aged individuals [12]. Youth and middle-aged individuals serve as a baseline for understanding changes in cognitive abilities that occur later in life. Studies suggest that identifying cognitive decline during middle age is necessary because the longer the time taken, the more neuronal degeneration would have progressed significantly [13]. As highlighted by Osler et al. [14] and Liu and Lee [15], there is a high incidence of cognitive decline in middle-aged adults with hearing loss. Studies concerning cognition in middle-aged adults with hearing loss have shown a decline in memory [4,16], language and processing speed [4]. Notably, recent findings suggest that individuals over 45 years of age with hearing loss are 1.69 times more likely to experience cognitive impairment than their counterparts with normal hearing [17].

Untreated hearing loss during midlife (45 years and above) has been recognized as the leading modifiable risk factor for dementia, contributing to approximately 7% of dementia cases [18]. Individuals with untreated hearing loss often experience cognitive decline, whereas treatment with amplification has been shown to improve cognitive outcomes [19]. The cognitive decline associated with hearing loss may be driven by increased cognitive load and reduced sensory stimulation [20]. Conversely, hearing aid use has been linked to enhanced cognitive performance by improving auditory input, reducing listening effort, and preserving cognitive resources [21]. Research further suggests that untreated hearing loss is associated with decreased activity in the frontal and prefrontal cortex. However, post-treatment findings indicate that use of hearing aids may lessen cognitive effort and modify top-down modulation of the auditory and visual cortices, potentially enhancing both auditory and visual processing [22]. Given these findings, studies investigating the impact of untreated hearing loss on cognition provide crucial evidence for counselling individuals on the benefits of amplification.

Despite the well-established link between untreated hearing loss and cognitive decline, its impact on cognition in young and middle-aged adults has not been systematically examined. A review of its impact on this population could inform clinical decisions, counselling, rehabilitation, and further research in those populations. While previous reviews have explored the relationship between hearing loss and cognition in older adults [8,23], a critical gap in the understanding of how untreated hearing loss affects cognition in younger populations remains. A meta-analysis by Taljaard et al. [19] comparing cognitive performance in adults with and without untreated hearing loss suggested a possible relationship between untreated hearing loss and cognitive decline; however, their conclusions were premature due to the heterogeneity of the included studies and the absence of strict inclusion criteria for age and amplification use. More recently, de Morais et al. [24] conducted a meta-analysis focused on adults and older adults, specifically comparing cognitive outcomes between hearing aid users and nonusers. In contrast, our review employs more rigorous inclusion and exclusion criteria while incorporating studies with pure tone audiometry assessment, age and amplification criteria to provide a more precise analysis. Given the limited number of systematic reviews exploring cognitive functions in adults with hearing loss, especially among younger and middle-aged populations, there is a clear need to better understand the cognitive impact of untreated hearing loss across various domains in this population. Therefore, the primary objective of this review is to address the following question: “Do young and middle-aged adults with untreated hearing loss have similar cognitive functions as young and middle-aged adults with normal hearing?”. It was hypothesized that young and middle-aged adults with untreated hearing loss would have reduced cognitive function compared with adults with normal hearing.

## 2. Materials and Methods

### 2.1. Data Sources and Searches

This study followed the guidelines of the Preferred Reporting Items for Systematic Reviews and Meta-Analyses (PRISMA) Statement. The inclusion and exclusion of studies were guided by the Population, Exposure, Comparison, Outcome, and Study design (PECOS) framework. A comprehensive and systematic search was conducted across four major electronic databases, MEDLINE (PubMed), Scopus, Web of Science, and EMBASE, from their inception to 30 May 2025. An additional search strategy was conducted via backward and forward reference searching of the eligible articles in the review. Keywords related to cognitive function in young and middle-aged adults with and without hearing loss were combined via appropriate Boolean operators. A detailed list of search terms and strategies used for each database is provided in Appendix A (Table A1). This review was registered in the International Prospective Register of Systematic Reviews (PROSPERO) on 21 February 2025, under the identifier CRD42025628016.

### 2.2. Study Selection

#### 2.2.1. Population

The review included studies focusing on young and middle-aged adults with and without hearing loss within an age range of 18–65 years. Studies focusing on adults with hearing loss of any type (conductive, sensorineural, or mixed) or severity (minimal to profound) were included. Only studies that reported peripheral hearing status via pure tone audiometry were considered. Studies including adults with acquired bilateral hearing loss due to aging, noise exposure, or other causes were also considered. Studies involving adults with congenital hearing loss or unilateral or sudden hearing loss or those relying on self-reported or undiagnosed hearing loss were excluded. Studies involving adults who used any form of amplification, those with self-reported neurogenic or psychogenic disorders, or those with uncorrected visual impairments were also excluded.

#### 2.2.2. Exposure

The review included studies that measured at least one cognitive function, either overall cognitive function or certain cognitive domains, such as memory, attention, or executive functions.

#### 2.2.3. Comparison

The review included comparisons of cognitive outcomes between adults with normal hearing and those with untreated hearing loss.

#### 2.2.4. Outcome

Studies that measured cognitive outcomes in hearing loss patients were included. The review included outcomes of different cognitive domains as investigated by any cognitive test in individuals with normal hearing and hearing loss (for example, working memory span on the basis of the digit span test and reaction time in processing speed on the Trail Making Test (TMT)).

#### 2.2.5. Study Designs

The review included all original human studies published in peer-reviewed English language journals, encompassing randomized controlled trials, case–control studies, cohort studies, and cross-sectional studies.

### 2.3. Data Extraction

All citations were imported, and duplicate records were identified and removed before beginning the screening process. Initially, two reviewers (LS and US) independently screened the titles and abstracts of the studies. Full texts of all remaining studies were reviewed independently by both reviewers to verify eligibility according to the predefined criteria. Any disagreements that occurred were resolved through discussion with a third reviewer (GB). Reasons for exclusion were recorded for any studies removed after the full-text review.

Data extraction was independently conducted by two reviewers (LS and US) via Excel. The ‘Data Collection Form’ template from the Cochrane Collaboration served as a guiding framework. For each included study, information was collected on general study details (author, year of publication, contact details, and country), study characteristics (study design, setting, and sample size), and data related to the PECOS framework. Discrepancies, if any, were addressed through discussion among the reviewers. Data extraction utilized a piloted form to ensure consistency. The extraction form was divided into six sections: general information, study eligibility, population and setting, methods, outcomes and findings, and additional information. On the basis of these details, a decision was made about whether to include or exclude the study. Finally, cognitive outcomes across all the domains for both groups were systematically extracted.

### 2.4. Risk of Bias Assessment

The methodological quality of all studies was independently assessed at the study level by two authors (L.S. and U.S.) via the Joanna Briggs Institute (JBI) Critical Appraisal Checklist for Analytical Cross-Sectional Studies [25]. This checklist has eight items evaluating key methodological elements, including sample selection criteria, study setting, exposure measurement, measurement of conditions, identification, control of confounding factors, measurement of outcomes, and statistical analysis. Each item is rated as ‘Yes,’ ‘No,’ ‘Unclear,’ or ‘Not applicable.’ Based on the proportion of ‘Yes’ ratings, studies were categorized by methodological quality as follows: strong (>70% ‘Yes’; moderate (50–69% ‘Yes’), and low (<49% ‘Yes’) [26].

### 2.5. Data Synthesis

All the included studies were qualitatively synthesized through tabulation according to the effect of hearing loss on cognition. The effects of hearing loss on cognition were categorized into four major cognitive domains (global cognitive function, memory, attention, and executive function). Eligible studies on the effects of hearing loss on cognition were included in the meta-analysis. The effect size (Cohen’s d) was computed from the reported mean and standard deviations using the Practical Meta-Analysis Effect Size Calculator [27]. If any study reported medians and interquartile ranges rather than means and standard deviations, the means and standard deviations were imputed following the approach proposed by Luo et al. [28]. In cases where a single study reported multiple relevant findings related to the research question (e.g., assessing the effect of hearing loss on different cognitive domains, such as memory and executive function), two different findings from the same study were reported.

All the meta-analyses were performed via JASP software (Version 0.19.3) [29]. The I^2^ statistic was used to assess heterogeneity among the included studies, expressed as a percentage ranging from 0–100%. It is classified into four categories: insignificant heterogeneity (0–25%), low heterogeneity (25–50%), moderate heterogeneity (50–75%) and high heterogeneity (>75%) [30]. Pooled effect sizes, along with their 95% confidence intervals (CIs), and *p* values, were calculated using a random-effects model. Effect sizes were categorized as trivial (<0.2), small (0.2–0.49), medium (0.5–0.79), or large (≥0.8) [31]. The significance level was set at *p* < 0.05. A forest plot was used to illustrate the results of individual studies and meta-analyses.

## 3. Results

### 3.1. Study Selection and Characteristics

**Figure 1:**
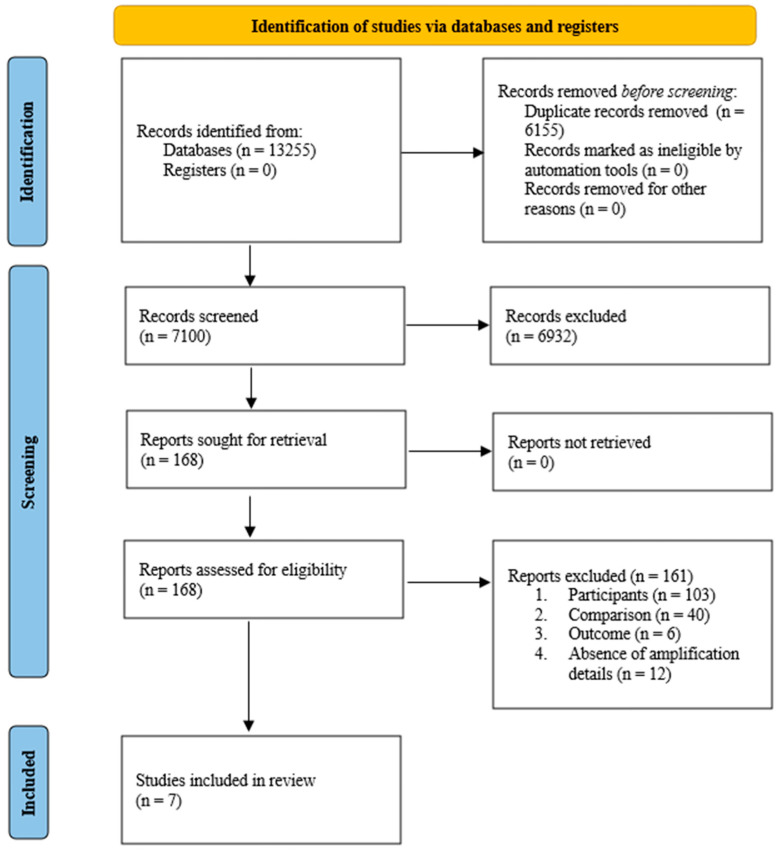
The Preferred Reporting Items for Systematic Reviews and Meta-Analyses (PRISMA) flow chart of the study identification, screening, eligibility, and inclusion process.

Among the 13,255 studies reviewed, a total of 7100 studies were screened on the basis of their titles and abstracts, resulting in the exclusion of 6155 studies before screening. Next, 168 full-text articles were assessed, 161 of which were excluded for the reasons outlined in Figure 1. Ultimately, seven studies were included in the qualitative synthesis, six of which were selected for the meta-analysis. Figure 1 depicts the PRISMA flow chart, outlining the process of identifying studies, screening records, assessing full-text eligibility, and determining the final set of included studies. Table 1 provides the characteristics of the studies included in the qualitative synthesis.

### 3.2. Risk of Bias Assessment

Across the included studies, the number of ‘Yes’ responses on the JBI checklist ranged from five to eight. Based on these ratings, one study was classified as having “moderate” methodological quality, whereas all other studies were rated “strong”. Detailed item-wise ratings for each study are summarized in Table 2. Importantly, no studies were excluded based on their methodological quality.

### 3.3. Narrative Synthesis

This systematic review synthesized findings from seven studies examining the impact of hearing loss on cognitive function in adults aged 18–65 years.

#### 3.3.1. Population

A total of seven studies involving 681 participants were included in this systematic review. Of these, 351 were individuals with hearing loss and 330 were individuals with normal hearing. The age of the participants ranged from 18–65 years, with a mean age of 50.38 ± 10.87 years in the hearing loss group and 46.64 ± 11.18 years in the control group. All included studies assessed hearing status via pure tone audiometry, ensuring objective and standardized classification of hearing loss. The type of hearing loss in all studies was sensorineural and acquired, with severity ranging from minimal to moderate. Among the six studies included in the meta-analysis, all studies except one [32] employed non-hearing aid users. Woods et al. [32] employed both hearing aid users and non-hearing aid users. However, only data from non-hearing aid users were included in the analysis to maintain consistency across studies.

#### 3.3.2. Cognitive Domains and Outcomes

The individual cognitive tests were grouped into four domains—global cognitive function, memory, executive function, and attention. Global cognitive function was assessed in one study, memory in four studies, executive function in five studies, and attention in one study. A study that assessed global cognition, via the Mini-Mental State Examination (MMSE) and the Montreal Cognitive Assessment (MoCA) [33], reported significantly lower scores in the hearing-impaired group than in the normal-hearing group, indicating a broad impact of hearing loss on general cognitive status. Within the memory domain, two out of four studies [4,34] reported significant deficits in memory performance among hearing-impaired individuals, particularly in tasks such as incidental learning, digit reconstruction, delayed word recall, and logical memory. However, Sharma et al. [35] and Woods et al. [32] reported no significant differences in tasks such as the Rey Auditory Verbal Learning Test (RAVLT), N-back, and digit span.

With respect to executive function, three out of five studies [32,36,37] reported statistically significant group differences in at least one executive task. Chandrashekar et al. [36] reported significant differences in TMT A and B, whereas Chandrashekar et al. [37] reported poorer performance in phonemic, semantic, and alternate fluency tasks among hearing-impaired participants. Woods et al. [32] identified differences in TMT B but not in A. In contrast, Sharma et al. [35] and Deal et al. [4] reported limited significance, with only the DSST showing notable group differences.

For attention, only one study [35] assessed both sustained and selective attention. Although no statistically significant group differences were reported for sustained attention (Digit vigilance task) or selective attention (Stroop task), individuals with hearing loss and tinnitus generally performed poorer than those with normal hearing and tinnitus. However, an exception was noted in the Digit Vigilance test error score, where a statistically significant difference emerged, with the group with normal hearing and tinnitus committing more errors than the group with hearing loss and tinnitus.

### 3.4. Meta-Analysis

A meta-analysis was performed to quantitatively synthesize findings from studies examining the relationship between untreated hearing loss and cognitive performance among young and middle-aged adults. Six studies were included in the overall meta-analysis assessing cognitive outcomes, whereas domain-specific analyses were performed separately for memory and executive function. Owing to the limited number of studies available for attention and global cognitive function, a meta-analysis of these domains was not performed.

#### 3.4.1. Effect of Hearing Loss on Overall Cognitive Performance

**Figure 2:**
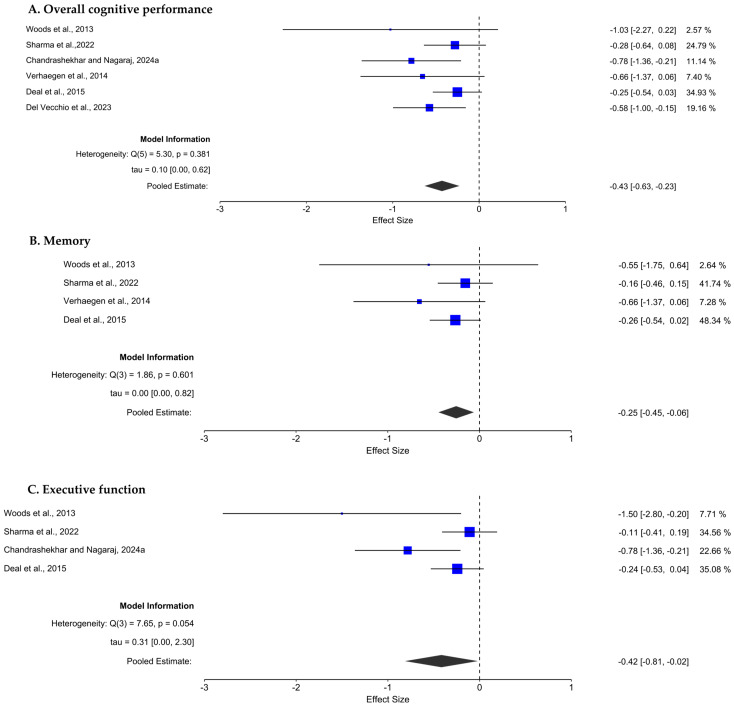
Forest plot depicting the effect of hearing loss on overall cognitive performance (**A**), memory (**B**) and executive function (**C**) [4,32,33,34,35,36].

The overall meta-analysis included six studies examining the broader impact of untreated hearing loss on cognitive function. The pooled analysis yielded a small but statistically significant effect size of −0.43 (95% CI: −0.63 to −0.23; *p* < 0.001), indicating that individuals with hearing loss performed worse on cognitive measures than their normal-hearing counterparts did. Heterogeneity among the studies was insignificant (14.25%), suggesting consistency across studies. A forest plot depicting the impact of hearing loss on overall cognitive performance is shown in Figure 2.

#### 3.4.2. Effect of Hearing Loss on Memory

To specifically investigate the impact of hearing loss on memory, four studies that assessed memory outcomes were included in a separate meta-analysis. The pooled effect size for memory performance was −0.25 (95% CI: −0.45 to −0.06; *p* = 0.010), indicating a small but statistically significant poorer memory performance in individuals with hearing loss than in those with normal hearing. A forest plot depicting the impact of hearing loss on memory is shown in Figure 2. Heterogeneity was insignificant (0%), suggesting that the studies were methodologically comparable and that the results were consistent. These findings suggest that hearing impairment may modestly impact memory function.

#### 3.4.3. Effect of Hearing Loss on Executive Function

A separate meta-analysis was also performed for executive function, including four studies. The pooled effect size was −0.42 (95% CI: −0.81 to −0.02; *p* = 0.040), demonstrating a small and statistically significant impact of hearing loss on executive function. The heterogeneity was moderate (66.76%), implying acceptable consistency among the included studies. A forest plot depicting the impact of hearing loss on executive function is shown in Figure 2. These results indicate that hearing loss may have a moderate adverse effect on executive functioning abilities such as planning, cognitive flexibility, and problem solving.

### 3.5. Sensitivity Analysis

To determine the contribution of each study to the overall pooled effect, a sensitivity analysis was performed in which studies were sequentially omitted and the meta-analysis recalculated. This analysis revealed similar effect sizes and statistical significance, even when each study was removed. Importantly, the direction and magnitude of the pooled effect size remained largely unchanged, indicating that the findings were stable and not unduly influenced by any single study. A summary of the sensitivity analyses performed is provided in Appendix B. Variations in the I^2^ statistic were observed, with some studies showing insignificant to low heterogeneity (0–28.68%) (Table A2 and Table A3) and others indicating moderate heterogeneity (I^2^ up to 74.40%) (Table A4). These fluctuations likely reflect differences in study design, participant characteristics, or outcome measures rather than instability in the overall findings.

## 4. Discussion

This systematic review and meta-analysis explored the cognitive effects of untreated hearing loss in young and middle-aged adults. Evidence from prior research has consistently reported associations between hearing loss and cognitive decline, particularly in older adults, across various domains, including memory, executive function, and global cognition [8,38]. Our findings suggest that similar cognitive vulnerabilities are also evident earlier in adulthood, although the effect size is small. The meta-analysis of overall cognitive performance across six studies revealed significantly poorer performance in adults with untreated hearing loss. This cognitive decline may be driven by prolonged sensory deprivation, which can lead to cortical volume reductions in brain regions involved in memory, executive control, and other higher-order functions [39]. Powell et al. [39] highlighted the challenges in data extraction from hearing and cognition studies, attributing them to the significant variability in methodologies and designs. There is a wide range of differences in audiometric and cognitive measures, the nature and size of the samples studied, and the approach to confounding factors, among other elements. However, the present meta-analysis had insignificant to moderate heterogeneity, which implies reasonably consistent findings among the studies included.

### 4.1. Effect of Hearing Loss on Memory

A small yet statistically significant effect was observed on memory performance in studies comparing individuals with and without untreated hearing loss. The brain regions most affected by hearing loss, such as the frontal and prefrontal cortices, superior temporal gyrus, and Heschl’s gyrus, are also critically involved in memory processes and may contribute to the trajectory of cognitive decline [40]. Notably, most of the studies included in this review assessed memory via tasks that relied on auditory input, which may have disproportionately impacted the performance of individuals with hearing loss due to impaired access to auditory input. This added perceptual challenge likely increases the cognitive load, as more effort is required to process degraded auditory signals, leaving fewer cognitive resources available for encoding and storing information [4]. As a result, working memory becomes burdened by the dual demands of perception and retention, aligning with the information degradation hypothesis, which suggests that the diversion of controlled and selective attention toward decoding auditory input compromises memory performance [4]. However, due to the limited number of studies included, the current review does not allow a meaningful comparison between auditory and non-auditory memory measures. This prevents a firm conclusion about whether the observed effects reflect perceptual degradation alone or broader cognitive mechanisms.

### 4.2. Effect of Hearing Loss on Executive Function

Executive function, involving tasks of processing speed and verbal fluency, demonstrated significantly poorer performance in adults with untreated hearing loss. This could be attributed to alterations in auditory input caused by peripheral hearing loss, which can disrupt neural pathways involved in cognitive control [41]. Executive functioning is primarily supported by frontal brain regions [42], and damage or reduced activation in the prefrontal cortex, which are frequently observed in individuals with hearing loss, may underlie these deficits [39] and may have contributed to this deficit in executive functioning. Notably, performance on the TMT, which requires cognitive flexibility and speed, has been strongly associated with speech-in-noise performance, which is diminished in the hearing loss population [43].

### 4.3. Effect of Hearing Loss on Global Cognition

Only one study in this review assessed the impact of hearing loss on global cognitive function. Del Vecchio et al. [33] reported that individuals with hearing loss presented significantly lower scores on global cognition measures than did their normal-hearing counterparts. The authors suggested that reduced auditory input may lead to abnormal stimulation and functional changes in both the auditory pathway and broader central nervous system, contributing to cognitive decline. Although these findings support the idea that hearing loss is associated with overall cognitive deterioration, the limited number of studies prevents strong conclusions and highlights the need for further research in this area.

### 4.4. Effect of Hearing Loss on Attention

The only study included in the review that assessed attention in individuals with untreated hearing loss [35] reported poor performance by individuals with hearing loss and tinnitus on both sustained and selective attention tasks, although these differences were not statistically significant. These findings align with existing evidence demonstrating reduced sustained attention [44] and altered selective attention [45] in individuals with hearing loss. Such attentional difficulties may arise from the increased cognitive effort required for degraded auditory input, leading to the faster depletion of attentional resources and a greater reliance on visual cues for both environmental awareness and task performance [44]. In contrast to these trends, the current study by Sharma et al. [35] observed significantly higher error scores in the digit vigilance task by individuals with normal hearing and tinnitus. This counterintuitive finding may be attributed to greater susceptibility to both environmental noise and the internal perceptual noise associated with tinnitus, which could have contributed to increased lapses in sustained visual attention in the normal hearing group.

Although most of the literature and clinical practice focus on cognitive decline and dementia in older adults with hearing loss, this review highlights that such cognitive vulnerabilities are also present in young and middle-aged adults. As emphasized by the Lancet Commission [18], hearing loss is a major modifiable risk factor for dementia beginning in midlife. Our findings reinforce the need for early cognitive screening in adults with untreated hearing loss to identify and address cognitive changes before they progress to more severe impairment or dementia. Despite this growing evidence, cognitive assessments are rarely included in routine audiological evaluations. It is imperative that hearing healthcare settings begin incorporating domain-specific cognitive screening tools as part of standard aural assessment and rehabilitation protocols. Early identification of cognitive deficits allows clinicians to tailor rehabilitation strategies accordingly and counsel patients on the cognitive benefits of timely hearing aid use or other interventions like cochlear implants, assistive listening devices, auditory training, communication strategy training and cognitive rehabilitation. Hence, by integrating cognitive aspects into routine audiological evaluation, hearing healthcare systems have the potential to delay or even prevent the future burden of Alzheimer’s disease and related dementia in the hearing loss population.

### 4.5. Limitations of the Study

This review has several limitations that should be acknowledged. First, the number of studies available for certain cognitive domains, particularly attention and global cognitive function, was limited, restricting the ability to conduct comprehensive domain-specific meta-analyses. Second, the predominance of cross-sectional designs among the included studies limits the ability to infer causal relationships between hearing loss and cognitive decline. Additionally, there was notable methodological variability across studies, including differences in the types of cognitive tasks used, modalities of test presentation (auditory vs. visual), cognitive domains considered in different studies, and participant characteristics such as age distribution and educational background. The duration and etiology of hearing loss are often not reported or standardized, making it difficult to compare findings across studies. Furthermore, many studies did not consistently account for potential confounding variables such as comorbidities, mental health status, lifestyle factors, or socioeconomic background and may have contributed to unexplained variability.

### 4.6. Future Implications

The findings from this review highlight several important directions for future research. Future studies should expand to include cognitive domains that remain underexplored, such as attention, visuospatial abilities and language, in young and middle-aged adults. It is also essential to investigate the role of hearing aids and other amplification devices in mitigating cognitive decline among younger and middle-aged adults, as most current evidence is based on older populations. Future studies should also place greater emphasis on systematically identifying and controlling for potential confounding variables, such as age, education, socioeconomic status, mental health, physical activity, and comorbid medical conditions, which can independently influence cognitive performance.

## 5. Conclusions

This review and meta-analysis explored the cognitive consequences of untreated mild-to-moderate hearing loss in young and middle-aged adults, a group underrepresented in cognitive hearing research. The findings from this review and meta-analysis revealed measurable declines in cognitive functioning, particularly in the memory and executive function domains. These results challenge the assumption that cognitive decline related to hearing loss is exclusive to older adults and highlight the need for early identification and intervention. The observed cognitive decline suggests that even mild-to-moderate hearing loss in midlife can affect neural and cognitive processing in ways that may have long-term consequences for brain health.

## Figures and Tables

**Table 1:** Characteristics of the included studies.

Study	Sample Size	Age (in Years), Mean ± SD	Pure Tone Average (dB HL)	Degree of Hearing Loss in HL Group	Cognitive Domains Tests Administered and Findings (‘+’ Indicates the HL Group Performed Significantly Poorer than the NH Group; ‘-’ Indicates the NH Group Performed Significantly Poorer than the HL Group, and ‘=’ Indicates no Significant Difference Between Groups)
Deal et al. [4]	Total = 253 NH = 73 Mild HL = 95 Moderate/severe HL = 85	NH: 53.8 ± 4 Mild HL: 56.4 ± 4.9 Moderate/severe HL: 59.1 ± 5.4	NH group 18.1 ± 6.3 HL group Mild HL: 33.2 ± 4.1 Moderate/severe HL: 49.6 ± 8.7	Mild to moderate	Memory DWRT: ‘+’ Incidental Learning Test: ‘+’ Logical Memory Test I and II: ‘=’ Digit Backward Test ‘=’ Boston Naming test: “=” Executive function Word fluency test: “=” Animal Naming test: “=” TMT A and B: “=” DSST: “+”
Woods et al. [32]	Total = 14 NH = 10 HL = 4	NH: 60 ± 12 HL: 36 ± 14	NH group RE: 5.4 ± 2.9 LE: 6.5 ± 3.4 HL group RE: 41.3 ± 4.6 LE: 41.2 ± 4.3	Mild to moderate	Memory Forward digit span recall: “=” Backward digit span recall: “=” Executive function TMT A: “=” TMT B: “+”
Del Vecchio et al. [33]	Total = 112 NH = 81 HL = 31	NH: 53.2 ± 4.8 HL: 58 ± 5.2	NH group RE: 12.5 ± 2.8 LE: 12.4 ± 3.1 HL group RE: 40.2 ± 18.7 LE: 41.2 ± 17.2	Mild to moderate	Overall cognition MMSE: “=” MoCA: “+”
Verhaegen et al. [34]	Total = 32 NH = 16 HL = 16	NH: 24.2 ± 1.4 HL: 25.2 ± 5.5	NH group 6.8 ± 3.7 HL group 17.2 ± 6.3	Minimal	Memory Immediate Serial Recall of words: “+” Immediate serial recall of phonologically similar and dissimilar words: “+” Digit serial order reconstruction: “+” Speeded nonword repetition: “=”
Sharma et al. [35]	Total = 170 NH = 100 HL = 70	NH: 37.4 ± 10.1 HL: 43.5 ± 9.7	NH group RE: 18.7 ± 3.2 LE: 18.9 ± 3.9 HL group PTA1 RE: 34.7 ± 10.5 LE: 37.4 ± 12.4 PTA2 RE: 50.6 + 17.9 LE: 58.2 + 14.5	Mild to moderate	Memory RAVLT “+” RCFT: “=” DST: “=” N back test: “=” Attention DVT (time taken): “=” DVT (error score): “-” Stroop test: “=” Executive function COWA: “=” Animal Names Test: “=” DSST: “+”
Chandrashekhar and Nagaraj [36]	Total = 50 NH = 25 HL = 25	NH: 49 ± 3.3 HL: 50.4 ± 2.8	NH group RE: 10.4 ± 4.0 LE: 10.6 ± 3.2 HL group RE: 33.3 ± 4.5 LE: 33.2 ± 4.2	Mild	Executive function TMT A and B: “+”
Chandrashekhar and Nagaraj [37]	Total = 50 NH = 25 HL = 25	NH: 49 ± 3.3 HL: 50.4 ± 2.8	NH group RE: 10.4 ± 4.0 LE: 10.6 ± 3.2 HL group RE: 33.2 ± 4.5 LE: 33.2 ± 4.2	Mild	Executive function Verbal fluency: “+”

Note. PTA1 is the average of pure-tone hearing thresholds at 500 Hz, 1 kHz and 2 kHz; PTA2 is the average of pure-tone hearing thresholds at 4 kHz, 8 kHz and 10 kHz; SD—Standard deviation; dB HL—decibel hearing level; NH—Normal Hearing; HL—Hearing Loss; RE—Right ear; LE—Left ear; MMSE—Mini Mental State Examination; MoCA—Montreal Cognitive Assessment; DWRT—Delayed Word Recall Test; DSST—Digit Symbol Substitution Test; TMT A—Trail Making Test Part A; TMT B—Trial Making Test Part B; RAVLT—Rey’s auditory verbal learning test; DST—Digit Span Test; RCFT—Rays complex figure test; DVT—Digit Vigilance test; COWA—Controlled oral word association test.

**Table 2:** Methodological quality appraisal of included articles.

Study	Were the Criteria for Inclusion in the Sample Clearly Defined?	Were the Study Subjects and the Setting Described in Detail?	Was the Exposure Measured in a Valid and Reliable Way?	Were Objective, Standard Criteria Used for Measurement of the Condition?	Were Confounding Factors Identified?	Were Strategies to Deal with Confounding Factors Stated?	Were the Outcomes Measured in a Valid and Reliable Way?	Was Appropriate Statistical Analysis Used?	Overall Appraisal	No. of Yes Responses	Methodological Quality
Del Vecchio et al. [33]	Yes	Unclear	Yes	Yes	Yes	Unclear	Yes	Yes	Include	6	Strong
Deal et al. [4]	Unclear	Unclear	Yes	Yes	Yes	Yes	Yes	Yes	Include	6	Strong
Verhaegen et al. [34]	Yes	Yes	Yes	Unclear	Yes	Unclear	Yes	Yes	Include	6	Strong
Chandrashekhar and Nagaraj [36]	Yes	Yes	Yes	Yes	Yes	Yes	Yes	Yes	Include	8	Strong
Chandrashekhar and Nagaraj [37]	Yes	Yes	Yes	Yes	Yes	Yes	Yes	Yes	Include	8	Strong
Sharma et al. [35]	Yes	Unclear	Yes	Yes	Yes	Unclear	Yes	Yes	Include	6	Strong
Woods et al. [32]	Yes	Unclear	Yes	Yes	Yes	Unclear	Unclear	Yes	Include	5	Moderate

## Data Availability

The datasets used and analysed during the current study are available from the corresponding author upon reasonable request.

## Abbreviations

The following abbreviations are used in this manuscript:

PRISMA	Preferred Reporting Items for Systematic Reviews and Meta-Analyses
PECOS	Population, Exposure, Comparison, Outcome, and Study design
PROSPERO	Prospective Register of Systematic Reviews
TMT	Trail Making Test
JBI	Joanna Briggs Institute
MMSE	Mini-Mental State Examination
MoCA	Montreal Cognitive Assessment
RAVLT	Rey Auditory Verbal Learning Test

## Appendix A

**Table A1:** Search strategy.

Database	Search String
Pubmed	((“young*”[Text Word] OR “middle aged”[Text Word] OR “midlife”[Text Word] OR “adult*” [Text Word]) AND (“hearing loss”[Title/Abstract] OR “hearing impairment”[Title/Abstract]) AND (“cogniti*”[Title/Abstract] OR “memory”[Title/Abstract] OR “executive function”[Title/Abstract] OR “verbal fluency”[Title/Abstract] OR “processing speed”[Title/Abstract])) AND ((humans[Filter]) AND (english[Filter]))
Scopus	(TITLE-ABS-KEY (((“young*” OR “middle-aged” OR “midlife” OR “adult”) AND (“hearing loss” OR “hearing impairment”) AND (“cogniti*” OR “memory” OR “executive function” OR “attention” OR “verbal fluency” OR “processing speed”)))) AND (LIMIT-TO (SUBJAREA, “MEDI”) OR LIMIT-TO (SUBJAREA, “NEUR”) OR LIMIT-TO (SUBJAREA, “HEAL”) OR LIMIT-TO (SUBJAREA, “PSYC”)) AND (LIMIT-TO (DOCTYPE, “ar”)) AND (LIMIT-TO (LANGUAGE, “English”))
Web of Science	TS = (((“young*” OR “middle aged” OR “midlife” OR “adult*”) AND (“hearing loss” OR “hearing impairment”) AND (“cogniti*” OR “memory” OR “executive function” OR “attention” OR “verbal fluency” OR “processing speed”))) and English (Languages) and Article (Document Types) and English (Languages)
Embase	(‘young*’:ti,ab,kw OR ‘middle aged’:ti,ab,kw OR ‘midlife’:ti,ab,kw OR ‘adult*’:ti,ab,kw) AND (‘hearing loss’:ti,ab,kw OR ‘hearing impairment’:ti,ab,kw) AND (‘cognition’:ti,ab,kw OR ‘cognitive’:ti,ab,kw OR ‘memory’:ti,ab,kw OR ‘executive function’:ti,ab,kw OR ‘attention’:ti,ab,kw OR ‘verbal fluency’:ti,ab,kw OR ‘processing speed’:ti,ab,kw)

## Appendix B

**Table A2:** The sensitivity analysis of all studies included in the meta-analyses: the impact of each study on the pooled estimate obtained after random-effects model meta-analyses for overall cognitive performance.

Study Omitted	Pooled Sample Size	Estimate	95% Confidence Interval		I^2^ (%)
			Lower Limits	Upper Limits	
Del Vecchio et al. [33]	524	−0.392	−0.613	−0.171	11.28
Deal et al. [4]	383	−0.524	−0.772	−0.276	9.43
Verhaegen et al. [34]	604	−0.411	−0.621	−0.202	15.42
Chandrashekhar and Nagaraj [36]	586	−0.369	−0.557	−0.181	0
Sharma et al. [35]	461	−0.512	−0.785	0.238	28.68
Woods et al. [32]	622	−0.409	−0.606	−0.212	11.54

The total sample size for overall cognitive performance was 636. The average pooled effect size of the sensitivity analysis for overall cognitive performance was 0.43, which is identical to the effect size of 0.43.

**Table A3:** The sensitivity analysis of all studies included in the meta-analyses: the impact of each study on the pooled estimate obtained after random-effects model meta-analyses for memory.

Study Omitted	Pooled Sample Size	Estimate	95% Confidence Interval		I^2^ (%)
			Lower Limits	Upper Limits	
Deal et al. [4]	221	−0.301	−0.672	−0.070	19.59
Verhaegen et al. [34]	442	−0.223	−0.424	−0.022	0
Sharma et al. [35]	299	−0.326	−0.580	0.072	0
Woods et al. [32]	460	−0.246	−0.443	−0.050	0.002

The total sample size for memory performance was 474. The average pooled effect size of the sensitivity analysis for memory performance was 0.27, which is almost identical to the effect size of 0.25.

**Table A4:** The sensitivity analysis of all studies included in the meta-analyses: the impact of each study on the pooled estimate obtained after random-effects model meta-analyses for executive function.

Study Omitted	Pooled Sample Size	Estimate	95% Confidence Interval		I^2^ (%)
			Lower Limits	Upper Limits	
Deal et al. [4]	239	−0.614	−1.319	−0.092	74.40
Chandrashekhar and Nagaraj [36]	442	−0.212	−0.418	−0.007	0
Sharma et al. [35]	317	−0.630	−1.222	−0.037	64.87
Woods et al. [32]	478	−0.293	−0.589	−0.003	50.22

The total sample size for executive function performance was 492. The average pooled effect size of the sensitivity analysis for executive function performance was 0.43, which is almost identical to the effect size of 0.42.
